# Inhibitory Effects of Antimicrobial Peptide JH-3 on *Salmonella enterica* Serovar Typhimurium Strain CVCC541 Infection-Induced Inflammatory Cytokine Release and Apoptosis in RAW264.7 Cells

**DOI:** 10.3390/molecules24030596

**Published:** 2019-02-07

**Authors:** Lei Wang, Xueqin Zhao, Xiaojing Xia, Chunling Zhu, Huihui Zhang, Wanhai Qin, Yanzhao Xu, Bolin Hang, Yawei Sun, Shijun Chen, Jinqing Jiang, Gaiping Zhang, Jianhe Hu

**Affiliations:** 1 College of Animal Science and Veterinary Medicine, Henan Institute of Science and Technology, Xinxiang 453003, China; wlei_007@163.com (L.W.); zxueqin0708@163.com (X.Z.); xiaojingxia_1@163.com (X.X.); chunlingzhu_001@126.com (C.Z.); huihuizhang_1@163.com (H.Z.); yanzhaoxu_1@163.com (Y.X.); bolinhang_1@163.com (B.H.); yaweisun_1@163.com (Y.S.); shijunchen_1@163.com (S.C.); jinqingjiang_1@163.com (J.J.); 2 College of Animal Science and Veterinary Medicine, Henan Agricultural University, Zhengzhou 450000, China; 3 Faculty of Veterinary Medicine, Sumy National Agrarian University, Sumy 40021, Ukraine; 4 Center for Experimental and Molecular Medicine, Academic Medical Center, University of Amsterdam, Meibergdreef 9, 1105 AZ, 1000 Amsterdam, The Netherlands; qwh0715@126.com

**Keywords:** antimicrobial peptide JH-3, apoptosis, cytokines, *Salmonella*

## Abstract

The antibiotic resistance of *Salmonella* has become increasingly serious due to the increased use of antibiotics, and antimicrobial peptides have been considered as an ideal antibiotic alternative. *Salmonella* can induce macrophage apoptosis and thus further damage the immune system. The antimicrobial peptide JH-3 has been shown to have a satisfactory anti-*Salmonella* effect in previous research, but its mechanism of action remains unknown. In this study, the effects of JH-3 on macrophages infected with *Salmonella* Typhimurium CVCC541 were evaluated at the cellular level. The results showed that JH-3 significantly alleviated the damage to macrophages caused by *S.* Typhi infection, reduced the release of lactic dehydrogenase (LDH), and killed the bacteria in macrophages. In addition, JH-3 decreased the phosphorylation level of p65 and the expression and secretion of interleukin 2 (IL-2), IL-6, and tumor necrosis factor-α (TNF-α) by inhibiting the activation of the mitogen-activated protein kinase (MAPK) (p38) signaling pathway and alleviating the cellular inflammatory response. From confocal laser scanning microscopy and flow cytometry assays, JH-3 was observed to inhibit the release of cytochrome c in the cytoplasm; the expression of TNF-αR2, caspase-9, and caspase-8; to further weaken caspase-3 activation; and to reduce the *S.*-Typhi-induced apoptosis of macrophages. In summary, the mechanism by which JH-3 inhibits *Salmonella* infection was systematically explored at the cellular level, laying the foundation for the development and utilization of JH-3 as a therapeutic alternative to antibiotics.

## 1. Introduction

*Salmonella* is a Gram-negative bacterium that primarily infects the gut and is an important zoonotic pathogen. *Salmonella* has nearly 2500 serotypes and mainly infects animals and humans, which results in high morbidity and mortality [1]. In addition, 93.8 million intestinal infections and 155,000 deaths are reported to be caused by *Salmonella* each year, and the majority of cases of bacterial food poisoning are caused by *Salmonella* [1,2]. With respect to animal husbandry, in 2015, up to a 29.2% isolation rate of *Salmonella* was observed in pig slaughterhouses in Hennan, China, among which *Salmonella* Typhimurium had the highest infection rate [3]. Salmonellosis causes great harm to the livestock and poultry industries, and *Salmonella* typically infects juvenile animals, causing sepsis, enteritis, and abortion. Animals that recover from *Salmonella* infection can carry the bacterium for a long time, which can lead to acute epidemic outbreaks under certain conditions, seriously affecting the sustainable development of animal husbandry [4]. Thus, the effective prevention and control of *Salmonella* infection is of great importance to the development of animal husbandry and public health [5]. Antimicrobial peptides (AMPs) are bioactive peptides encoded by host genes and are the first line of defense against pathogen invasion. AMPs are key components of the host innate immune system and possess excellent activities against disease, including antibacterial, antiviral, antifungal, antiparasitic, and anticancer activities [6,7] AMPs are widespread in organisms and have been isolated from a wide range of species, including single-celled microbes, insects, invertebrates, plants, amphibians, birds, fish, mammals, and humans. AMPs are typically composed of 12–100 amino acids and are relatively short and amphiphilic, with most carrying two to nine positive charges [8]. AMPs with small molecular weights have the advantages of good solubility, strong thermal stability, a broad spectrum of antibacterial activity, and availability from a wide range of source materials. In addition, AMPs possess unique antibacterial mechanisms and have ideal antimicrobial activities against clinical multidrug-resistant strains without inducing bacterial resistance [9]. To date, more than 2000 types of AMPs have been identified [10]. Owing to the advantages described above, AMPs are expected to become one of the most promising alternatives to antibiotics.

In a previous study, we showed that the antimicrobial peptide JH-3 exhibited a broad spectrum of antibacterial activity and had particularly excellent antibacterial activity against *Salmonella* (CVCC541), *Escherichia coli* (ATCC25922), *Staphylococcus aureus* (ATCC29213), and *Candida albicans* (90029). Furthermore, JH-3 had notable protective effects in mice infected with a lethal dose of *Salmonella* [11]. Macrophages play a dual role in host invasion by *Salmonella*. In addition to their role in the immune response, macrophages also act as host cells of *Salmonella*, promoting the spread throughout the body and resulting in infection [12]. The aim of the present study was to elucidate the effects of JH-3 on the invasion of macrophages by *Salmonella enterica* Serovar Typhimurium strain CVCC541. The results indicated that JH-3 can significantly reduce the damage to macrophages caused by *Salmonella* CVCC541; shorten the survival time of *Salmonella* CVCC541 in macrophages; decrease the *Salmonella*-CVCC541-mediated release of the inflammatory cytokines interleukin 2 (IL-2), IL-6, and tumor necrosis factor-α (TNF-α); and inhibit apoptosis. In this study, the mechanism by which JH-3 inhibits *Salmonella* infection was systematically studied at the cellular level, laying the foundation for the application, development, and future research of the immunomodulatory effects of JH-3.

## 2. Results

### 2.1. High Expression of Inflammatory Cytokines by Macrophage RAW264.7 Cells Was Induced by *Salmonella* CVCC541 Infection

**Figure 1:**
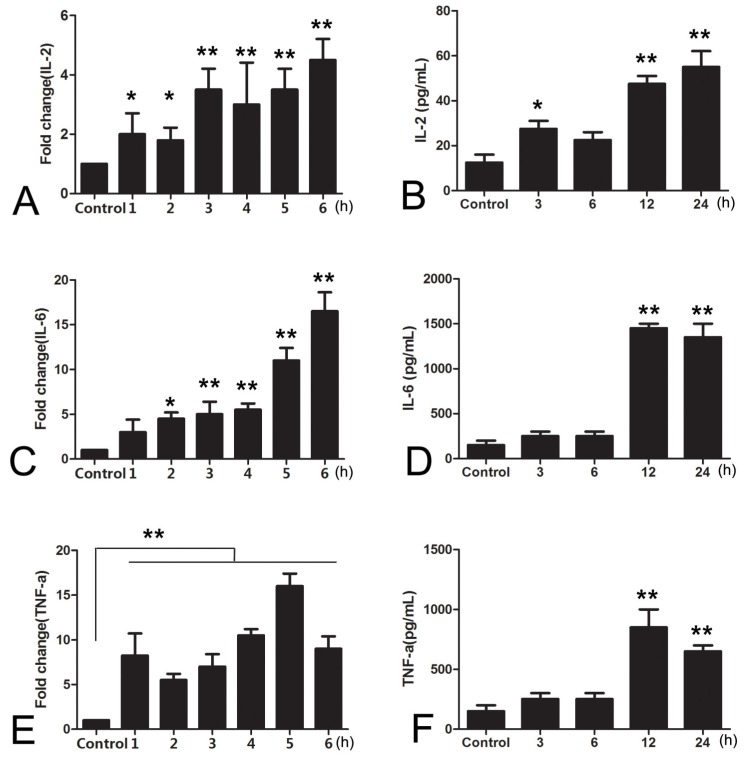
The expression of inflammatory cytokines induced by the infection of RAW264.7 cells by *Salmonella enterica* Serovar Typhimurium strain CVCC541. (**A**,**C**,**E**): the mRNA expression levels of interleukin 2 (IL-2), IL-6, and tumor necrosis factor-α (TNF-α) after RAW264.7 cells were infected with *Salmonella* CVCC541 (“*”, *p* < 0.05; “**”, *p* < 0.01 ); (**B**,**D**,**F**): the ELISA results of IL-2, IL-6, and TNF-α expression levels after RAW264.7 cells were infected with *Salmonella* CVCC541 (“*”, *p* < 0.05; “**”, *p* < 0.01).

Inflammation is accompanied by the production of inflammatory cytokines, such as TNF-α, IL-2, and IL-6 [13]. To evaluate the effects of *Salmonella* CVCC541 infection on the expression of inflammatory cytokines, the levels of TNF-α, IL-2, and IL-6 were assessed via RT-PCR and ELISA at different time points after the infection of RAW264.7 cells with CVCC541. The mRNA expression level of both IL-2 and IL-6 was notably increased after 1 and 2 h of infection and was further dramatically enhanced after 6 h of infection (*p* < 0.01, Figure 1A,C). Moreover, the mRNA expression level of TNF-α was elevated significantly from 1 to 6 h after infection, which was in stark contrast to that observed in the negative control group (*p* < 0.01, Figure 1E). In the ELISA assays, IL-2 was significantly upregulated at the early stage (2 h) after infection (*p* < 0.05, Figure 1B), and the peak IL-2 concentration (at 24 h) was notably greater than that observed in the control (*p* < 0.015, Figure 1B). In addition, the concentrations of IL-6 and TNF-α were not significantly different from those observed in the negative control group, reaching maximum values at 12 h (*p* < 0.01, Figure 1D,F), after which they decreased. These results showed that the expression of inflammatory cytokines in RAW264.7 cells could be significantly upregulated via *Salmonella* CVCC541 infection. In addition, these results showed that the infection of RAW264.7 cells by *Salmonella* CVCC541 could be used as an inflammatory cell model system.

### 2.2. Apoptosis of RAW264.7 Cells Was Induced by *Salmonella* CVCC541 Infection

**Figure 2:**
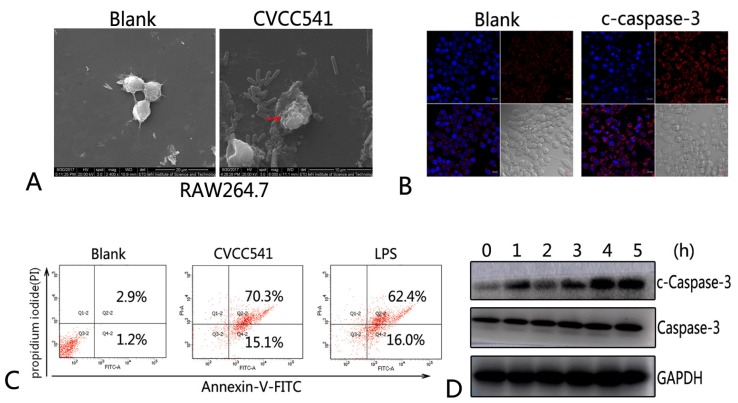
The apoptosis of RAW264.7 cells was induced by *Salmonella* CVCC541 infection. (**A**) The RAW264.7 cells infected with *Salmonella* CVCC541 were observed by scanning electron microscopy. (**B**) The c-caspase-3 activation in RAW264.7 cells was detected by confocal laser scanning microscopy. (**C**) The apoptosis rate of the infected RAW264.7 cells was detected by flow cytometry. (**D**) The caspase-3 and c-caspase-3 activations in RAW264.7 cells were detected by western blot (WB).

To explore the effects of *Salmonella* CVCC541 infection on apoptosis, RAW264.7 cells were infected with *Salmonella* CVCC541 (multiplicity of infection (MOI) = 10), after which the cell morphology was observed by scanning electron microscopy and the apoptosis rate of the infected RAW264.7 cells was assessed by flow cytometry. Apoptosis of the RAW264.7 cells could be observed after infection by *Salmonella* CVCC541, including cell membrane blebbing, cell shrinkage, chromatin condensation, and the formation of apoptotic bodies (indicated by a red arrow in Figure 2A). However, the surface of the negative control cells was smooth and round, and the cell morphology was complete (Figure 2A). In addition, the apoptosis rate of the infected RAW264.7 cells was notably increased by both *Salmonella* CVCC541 infection and lipopolysaccharides (LPS) treatment (2.9% vs. 70.3% for CVCC541, 2.9% vs. 62.4% for LPS, Figure 2C). Next, confocal laser scanning microscopy assays and Western blot analysis were conducted. As shown in Figure 2B, c-caspase-3 activation began 1 h after infection, and the level of activated c-caspase-3 was significantly increased upon entering the cell nucleus. Moreover, there was a positive correlation between its activation degree and the duration of infection (Figure 2D). The results indicated that the apoptosis of RAW264.7 cells could be induced by *Salmonella* CVCC541 infection.

### 2.3. JH-3 Significantly Inhibited the Expression of Inflammatory Cytokines

**Figure 3:**
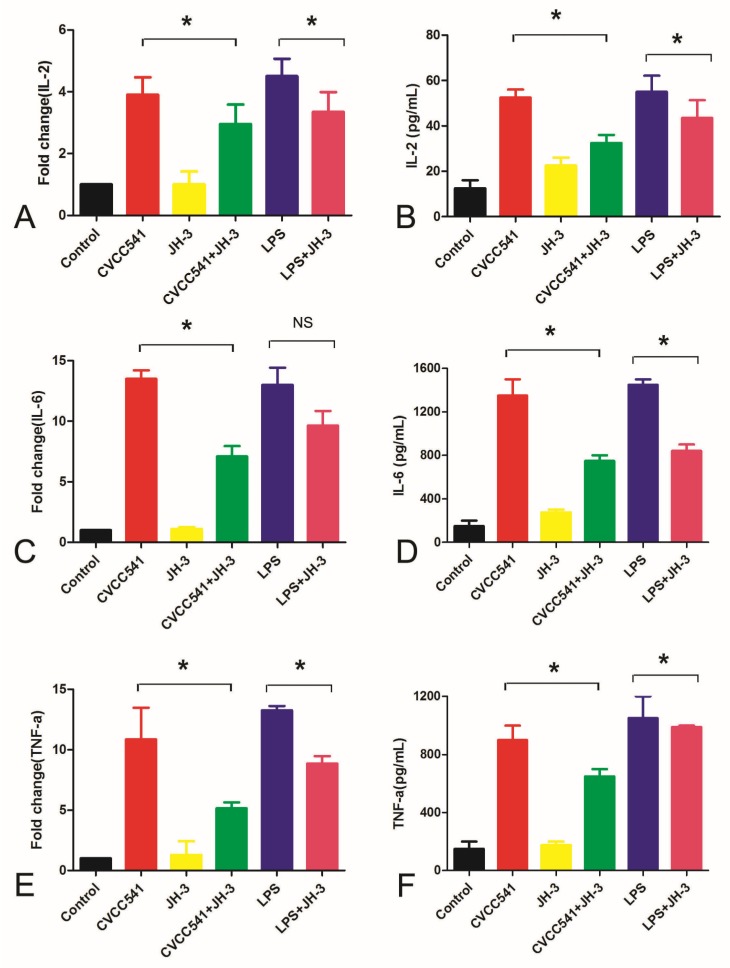
JH-3 inhibited the expression of inflammatory cytokines. (**A**), (**C**), and (**E**): JH-3 inhibited the mRNA expression of IL-2, IL-6, and TNF-α by RAW264.7 cells infected with *Salmonella* CVCC54 (“NS”, *p* > 0.05; “*”, *p* < 0.05). (**B**), (**D**), and (**F**): JH-3 inhibited the secretion of IL-2, IL-6, and TNF-α by RAW264.7 cells infected with *Salmonella* CVCC541 (“NS”, *p* > 0.05; “*”, *p* < 0.05).

As shown in the above findings, *Salmonella* CVCC541 infection of RAW264.7 cells could induce high inflammatory cytokine expression. To investigate the anti-inflammatory effects of JH-3, the expression of IL-2, IL-6, and TNF-α was determined via qRT-PCR and ELISA after the treatment of RAW264.7 cells with JH-3. The RAW264.7 cells were pretreated with JH-3 for 1 h and infected with *Salmonella* CVCC541 at an MOI of 10 or were treated with LPS as the positive control. The results indicated that the mRNA expression of IL-2, IL-6, and TNF-α induced by *Salmonella* CVCC541 infection was significantly inhibited after the JH-3 treatment (*p* < 0.05, Figure 3A,C,E). At the same time, the mRNA expression of IL-2 and TNF-α caused by LPS was also greatly reduced by the JH-3 treatment (*p* < 0.05, Figure 3A,E), while the effects of JH-3 on the expression of IL-6 caused by the LPS treatment was not significantly altered (*p* > 0.05, Figure 3C). Moreover, JH-3 could also significantly reduce the secretion of IL-2, IL-6, and TNF-α produced via the induction of macrophages by *Salmonella* CVCC541 and LPS (*p* < 0.05, Figure 3B,D,F). The results suggested that JH-3 could notably inhibit *Salmonella*-CVCC541-induced inflammatory cytokine expression in RAW264.7 cells.

### 2.4. JH-3 Inhibited the Activation of the Mitogen-Activated Protein Kinase (MAPK) and P65 Signaling Pathways

**Figure 4:**
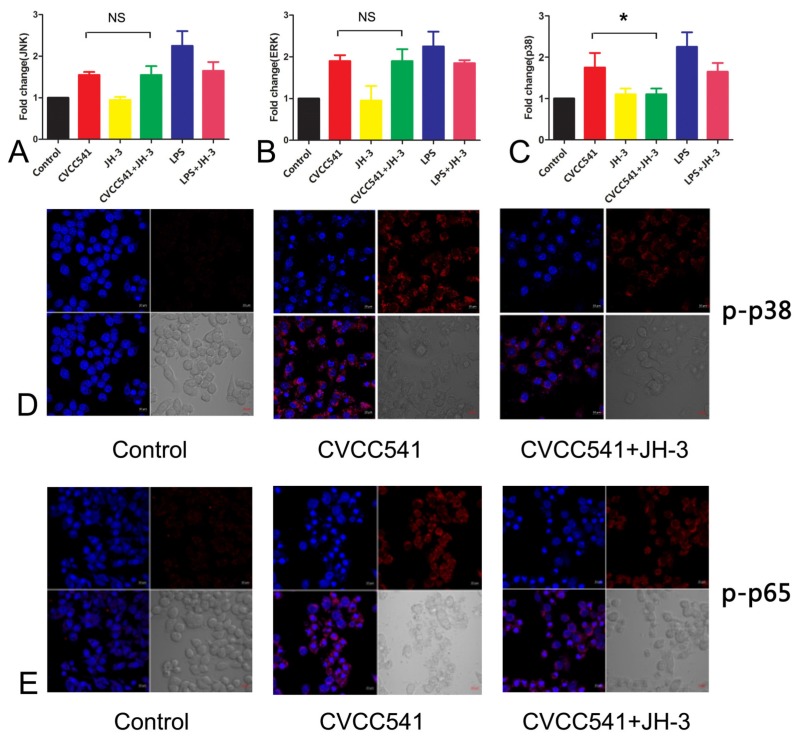
JH-3 inhibited the activation of the mitogen-activated protein kinase (MAPK) and p65 signaling pathways. (**A**–**C**): the effect of JH-3 on the mRNA expression level of JNK, ERK, and p38 assessed by qRT-PCR (“NS”, *p* > 0.05; “*”, *p* < 0.05). (**D**,**E**): the effect of JH-3 on the expression of p-p38 and p-p65 in RAW264.7 cells assessed by confocal laser scanning microscopy assays.

Mitogen-activated protein kinases are a group of serine/threonine protein kinases that are activated by multiple extracellular signaling pathways and are the final step of cytoplasmic signal transduction pathways. These proteins can be transferred into a cell nucleus after activation and play a role in the activation of nuclear transcription factor p65, regulating gene expression and taking part in cytokine secretion and apoptosis [14,15]. The results of the present study showed that JH-3 notably reduced p38 mRNA expression compared to the untreated group infected with *Salmonella* CVCC541 (*p* < 0.05, Figure 4C). However, JH-3 had no effect on the mRNA expression of JNK and ERK (*p* > 0.05, Figure 4A,B). In addition, JH-3 also inhibited LPS-induced mRNA expression of JNK, ERK, and P38 to different degrees. Through confocal laser scanning microscopy assays, JH-3 was further confirmed to significantly decrease the phosphorylation of p38 and p-p65 (Figure 4D,E), indicating that JH-3 could reduce the phosphorylation of p38 and the activation of p65, thus inhibiting the release of inflammatory cytokines.

### 2.5. JH-3 Reduced the Release of Lactic Dehydrogenase (LDH) and the Survival of Bacteria in RAW264.7 Cells

**Figure 5:**
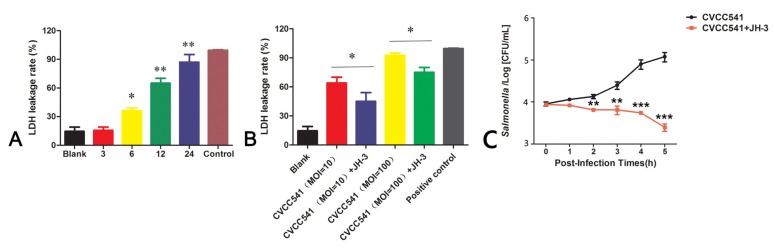
JH-3 reduced the release of lactic dehydrogenase (LDH) and the survival of bacteria in RAW264.7 cells. (**A**) The LDH release by RAW264.7 cells at different infection times (“*”, *p* < 0.05; “**”, *p* < 0.01). (**B**) JH-3 could inhibit LDH release by RAW264.7 cells at different multiplicities of infection (MOIs) (“*”, *p* < 0.05). (**C**) JH-3 could significantly inhibit the proliferation of CVCC541 and promote the killing of intracellular bacteria (“**”, *p* < 0.01; “***”, *p* < 0.001).

The release of LDH by RAW264.7 cells was monitored at the different time points after *Salmonella* CVCC541 infection (MOI = 10). LDH levels were remarkably increased, especially 12 and 24 h after infection, suggesting that cell membrane damage was gradually enhanced as the *Salmonella* CVCC541 infection progressed (*p* < 0.05 for 6 h and *p* < 0.01 for 12 and 24 h, Figure 5A). However, the release of LDH was notably reduced when the RAW264.7 cells were pretreated with JH-3 for 1 h prior to being infected with *Salmonella* CVCC541 at an MOI of 10 or 100 (*p* < 0.05, Figure 5B). Furthermore, the addition of 1 minimum inhibitory concentration (MIC) of JH-3 to the cell culture medium was observed to significantly inhibit the proliferation of *Salmonella* CVCC541, as assessed by bacterial counting, and played a role in killing intracellular bacteria (*p* < 0.01 and *p* < 0.001, Figure 5C).

### 2.6. JH-3 Inhibited the *Salmonella*-CVCC541-Induced Apoptosis of RAW264.7 Cells

**Figure 6:**
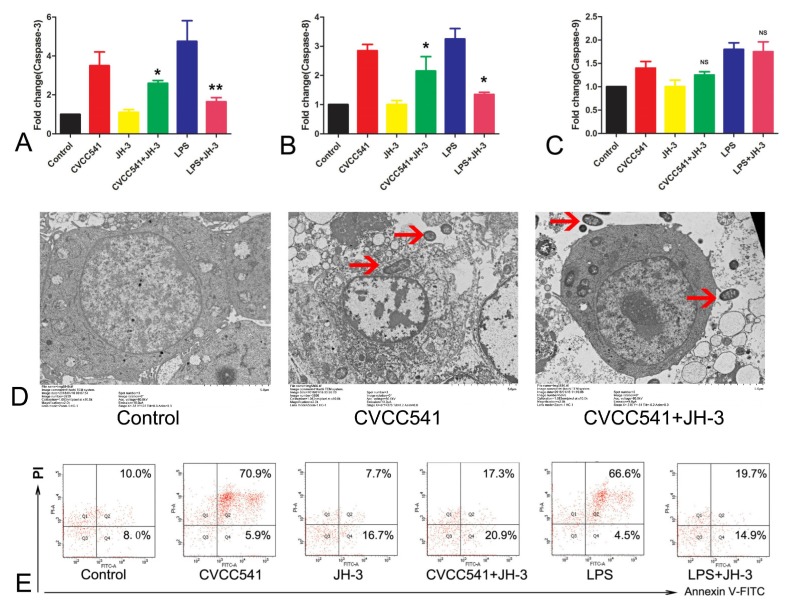
JH-3 inhibited *Salmonella*-CVCC541-induced apoptosis in RAW264.7 cells. (**A**–**C**): the effect of JH-3 on the mRNA expression of caspase-3, caspase-8, and caspase-9 (“NS”, *p* > 0.05; “*”, *p* < 0.05; “**”, *p* < 0.01). (**D**): the effect of JH-3 on cell morphological structure as assessed by transmission electron microscopy. (**E**): JH-3 reduced the apoptosis rate of RAW264.7 cells as assessed by flow cytometry analysis.

Previous studies showed that *Salmonella* CVCC541 infection can induce the apoptosis of RAW264.7 cells. To evaluate the effects of JH-3 on *Salmonella*-CVCC541-induced apoptosis, apoptosis was evaluated by qRT-PCR, transmission electron microscopy, and flow cytometry. JH-3 notably decreased the expression of caspase-3 (*p* < 0.05, Figure 6A) and caspase-8 (*p* < 0.05, Figure 6B), indicating that JH-3 could disrupt apoptosis by inhibiting the activation of exogenous signaling pathways. Moreover, JH-3 also reduced the expression of the caspase-9 gene, although the difference was not significant (*p* = 0.07, Figure 6C). Incomplete cell morphological structure, severely damaged cell membranes, condensed and marginalized cell nuclei, and apoptotic bodies were observed in the *Salmonella* CVCC541 infection group by TEM (the middle graph in Figure 6D; the red arrow indicates *Salmonella* CVCC541). In the JH-3 treatment group, relatively complete cell structures, few apoptotic bodies, and normal cell nuclei could be observed, suggesting that JH-3 enhanced the ability of cells to fight the bacteria (the right graph in Figure 6D; the red arrow shows *Salmonella* CVCC541). In the negative control group, the cytoplasm was uniform, the nucleus was round, and the cell structure was complete (the left graph in Figure 6D). In the flow cytometry analysis, JH-3 was observed to significantly reduce the apoptosis rate of RAW264.7 cells by more than threefold (Figure 6E, 17.3% vs. 70.9% for *Salmonella* CVCC541, 19.7 vs. 66.6% for LPS). Thus, the above results demonstrate that JH-3 could inhibit the apoptosis induced by *Salmonella* CVCC541 and LPS.

### 2.7. JH-3 Downregulated the Expression of Caspase-9 and TLR4

**Figure 7:**
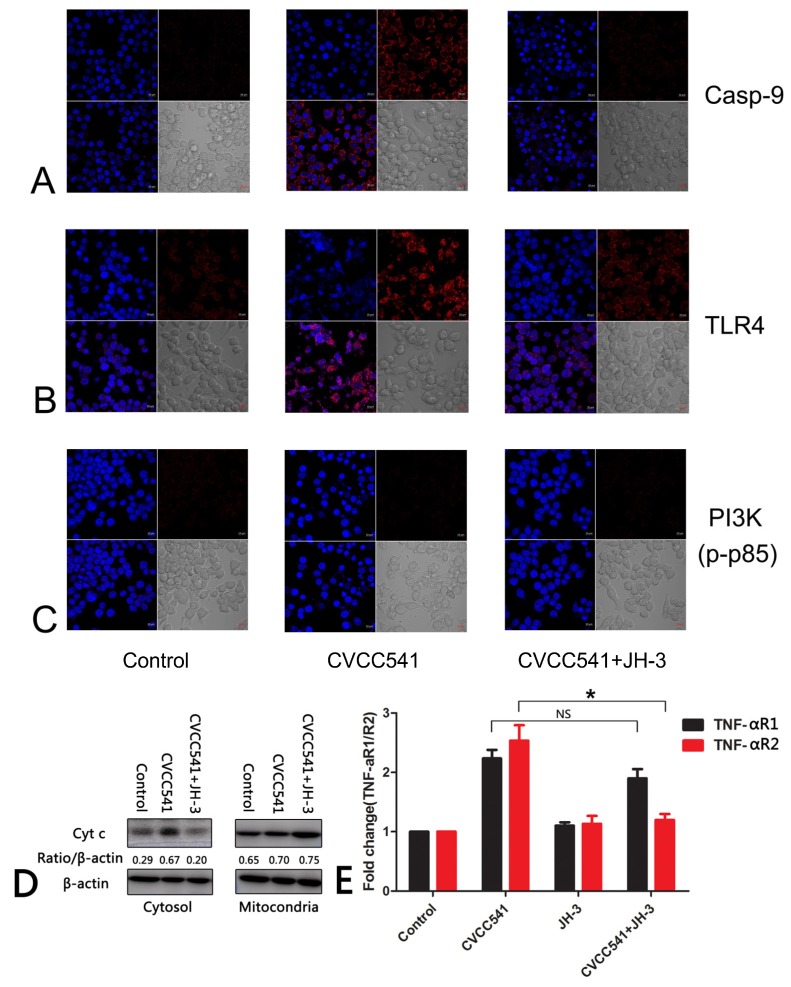
JH-3 downregulated the expression of caspase-9 and TLR4. (**A**) The expression of caspase-9 in RAW264.7 cells assessed by confocal laser scanning microscopy. (**B**) The expression of TLR4 in RAW264.7 cells assessed by confocal laser scanning microscopy. (**C**) The expression of p-p85 in RAW264.7 cells assessed by confocal laser scanning microscopy. (**D**) JH-3 could significantly reduce the release of cytochrome c in the cytoplasm. (**E**) JH-3 could also reduce the expression of TNF-αR2 (“NS”, *p* > 0.05; “*”, *p* < 0.05).

The confocal laser scanning microscopy assays showed that JH-3 effectively reduced the protein expression levels of caspase-9, indicating that JH-3 inhibited endogenous apoptosis pathways (Figure 7A). The expression of TLR4 in RAW264.7 cells was upregulated after *Salmonella* CVCC541 infection, which could be effectively inhibited by JH-3 treatment, demonstrating a connection between JH-3 and TLR4, although the associated mechanism remains unclear. The PI3K cell pathway was not activated in either the *Salmonella* CVCC541 infection and or JH-3 treatment groups, suggesting that the PI3K pathway was not involved in *Salmonella* CVCC541 infection or the mechanism by which JH-3 inhibits *Salmonella* infection (Figure 7C). The release of cytochrome c in the cytoplasm and mitochondria was detected by Western blot. The results showed that cell JH-3 could significantly reduce the release of cytochrome c in the cytoplasm (Figure 7D). We used qRT-PCR to detect the expression of the TNF-α receptors (R2 and R1) and found that the expression of TNF-αR1 and TNF-αR2 were upregulated in the cells with CVCC541 infection. However, the expression of TNF-αR2 (not TNF-αR1) was downregulated when RAW264.7 cells were pretreated with JH-3 and then infected with CVCC541 (*p* < 0.05, Figure 7E)

## 3. Discussion

JH-3 is currently isolated in our lab from bovine spleens and involves manual optimization, and its antimicrobial activity and spectrum are higher than the originally described peptide [11]. JH-3 has been shown to significantly inhibit the lethality of *Salmonella* in mice and alleviate *Salmonella*-associated intestinal inflammation. As cells of the innate immune system, macrophages play an important role in the prevention of pathogen invasion. However, the mechanism by which JH-3 inhibits *Salmonella* invasion of macrophages remains unknown and to be elucidated. In the present study, JH-3 was observed to reduce *Salmonella*-CVCC541-induced secretion of IL-2, IL-6, and TNF-α and inhibit the cellular inflammatory response. JH-3 could also reduce *Salmonella*-induced RAW264.7 cell apoptosis by inhibiting the release of cytochrome c in the cytoplasm, the expression of TNF-αR2, and the activation of caspase-9 and caspase-8, thus reducing cell apoptosis induced by *Salmonella*. The functional mechanism of JH-3 was first explored in this study to lay a theoretical foundation for the future research and development of new JH-3 antimicrobial drugs.

The anti-inflammatory activity of AMPs has become a research hotspot in recent years. KR-12-a5 is a 12-meric α-helical AMP with a function similar to LL-37. A previous study showed that KR-12-a5 and its analogues can significantly inhibit the secretion of NO, TNF-α, IL-6, and MCP-1 from LPS-stimulated RAW264.7 cells, thus inhibiting the cellular inflammatory response [16]. Moreover, Han et al. observed a reduction in the expression of IL-6, IL-1β, TNF-α, and TLR2 and in the secretion of the proinflammatory cytokines IL-8, TNF-α, MMP-2, and TLR2, and the inflammatory response in a rat ear model was weakened when HaCaT cells and human monocytes were treated with different concentrations of AMP CEN1HC-Br after stimulation by *Propionibacterium acnes.* In addition, the anti-inflammatory effect of CEN1HC-Br was better than that of clindamycin [17]. In a study on the modulatory effect of analogue intestinal peptide rVIPa on trinitrobenzene sulfonic acid (TNBS)-induced colitis in rats, it was demonstrated that rVIPa could significantly reduce the expression of rabbit colonic TNF-α, increase the content of colonic IL-10, and alleviate inflammation of the colon in rabbits [18]. Fusco et al. showed that AMPs hBD-2 and hBD-3 inhibited the expression of the proinflammatory cytokines IL-6, IL-8, TNF-α, and IL-1β and promoted the expression of anti-inflammatory cytokine TGF-β after *Salmonella* infection in Caco-2 cells, demonstrating that AMPs can reduce the inflammatory response caused by infection [19]. A study by Wang et al. indicated that AWRK6 offered satisfactory protection against endotoxemia, promoting reductions in the serum levels of IL-1β, IL-6, and TNF-α while also reducing the inflammatory response in a murine endotoxemia model [20]. In this study, we showed that JH-3 decreased the release of the inflammatory cytokines IL-2, IL-6, and TNF-α by inhibiting the MAPK (p38) signaling pathway, demonstrating that JH-3 also had a satisfactory anti-inflammatory effect, which had been indicated in previous reports [16,18,20].

The function of AMP is closely related to the MAPK and NF-κB signaling pathways. In a study by Nijnik et al, AMP IDR-1002 was observed to provide protection against *E. coli* infection and induce the secretion of the human peripheral blood monocytes chemokines CXCL1, CXCL2, CXCL7, and CXCL8 via a Gi-coupled receptor as well as the PI3K, NF-κB, and MAPK signaling pathways [21]. Kogut et al. discovered that AMP BT downregulated the expression of proinflammatory cytokines (IL-1β and IL-6) and inflammatory chemokines (CXCLi1 and CXCLi2) by inhibiting the phosphorylation levels of proteins in the MAPK signaling pathway [22]. Moreover, LL-37 treatment enhanced the proliferation and migration of adipose-derived stromal/stem cells (ASCs) by activating MAPK, upregulating EGR1 expression, and promoting hair growth [23]. The AMPs myxinidin2 and myxinidin3 were shown to facilitate healing in infected wounds by decreasing inflammation through the regulation of downstream mediators, such as STAT3, p38, JNK, and EGFR [24]. Weber et al. showed that a truncated version of LL-37 significantly reduced the number of cancer cells via competitive inhibition of MAPK phosphorylation, indicating that the anticancer effect of LL-37 was dependent on the MAPK signaling pathway [25]. Furthermore, LL-37 could also promote the differentiation of epithelial and smooth-muscle-like markers through activation of the NF-κB signal transduction pathway [26]. According to the results obtained in the present study, we showed that the activity of JH-3 was associated with the P38 MAPK and NF-κB signaling pathways. Furthermore, JH-3 could inhibit p65 subunit activation by inhibiting P38 expression, reducing the secretion of the inflammatory cytokines IL-2, IL-6, and TNF-α.

AMPs have been shown to promote the apoptosis of tumor cells [27,28,29], and a melittin derivative (TT-1) was shown to stimulate caspase-3, caspase-9, and Bax expression and promote the apoptosis of human thyroid cancer cells (TT) following TT-1 treatment [29]. To improve the specificity and penetration of anticancer peptides against tumors, the AMPs HPRP-A1 and iRGD were coadminstered to tumor cells to destroy cell membranes, adhere to mitochondria, and induce apoptosis via a caspase-dependent pathway [27]. Low concentrations of the AMP MSP-4 were shown to induce the apoptosis of osteosarcoma MG63 cells through Fas/FasL- and a mitochondria-mediated pathway, which could be reversed by caspase-8 and caspase-9 inhibitors, suggesting that MSP-4 can be a potentially alternative drug for the treatment of human osteosarcoma [28]. Caspase-8 was activated after 24 h of treatment in chronic myeloid leukemia cells (K562) with the AMP PaDef, whereas caspase-9 was not activated, suggesting that PaDef-induced apoptosis, which was related to caspase-8 activation, was primarily caused by the activation of exogenous signaling pathways [5]. However, in this study, we observed that JH-3 could inhibit the *Salmonella*-induced apoptosis of RAW264.7 cells, the reason for which may be that *Salmonella* can promote the apoptosis of macrophages [30,31]. Invasive *Salmonella* have been reported to induce the apoptosis of macrophages as a part of the infection process, which may allow it to avoid detection by the innate immune system [32]. In addition, AMP PR39 has potent antioxidant effects that may prolong the survival of cells during hypoxia and reduce the apoptosis rate under hypoxic conditions [33]. In this study, *S.* Typhi increased the expression of Cyt C protein level in the cytoplasm, and Cyt C could be involved in activation of caspase-3. JH-3 inhibited this effect of *S.* Typhi in the cytoplasm and the apoptosis caused by *Salmonella*. Thus, JH-3 can inhibit the apoptosis of macrophages, increasing the host defense to inhibit bacterial infection.

**Figure 8:**
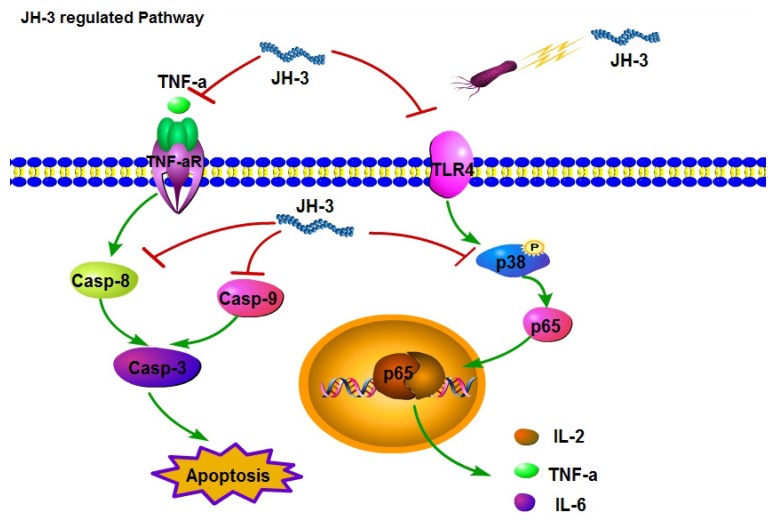
Signaling pathways regulated by antimicrobial peptide JH-3. On the left side of the figure, the inhibitory effect of JH-3 on the expression of TNF-αR2, caspase-8 (Casp-8), and caspase-9 (Casp-9) is shown. These changes inhibit the apoptosis of *Salmonella*-infected macrophages. On the right side of the figure, the inhibitory effect of JH-3 on the proliferation of *Salmonella* CVCC541 is shown (top), as well as the downregulation of TLR4 and the activity of p38 (MAPK) by JH-3 (bottom). The latter changes may inhibit cytokine production by *Salmonella*-infected macrophages.

In summary, *Salmonella*-induced apoptosis of macrophages is known to be accompanied by the activation of caspase-3 and an increase in the inflammatory cytokines TNF-α, IL-6, and IL-1β [32,34]. In this study, we demonstrated that JH-3 decreased *Salmonella* CVCC451 growth and LPS content, inhibited bacterial-infection-induced TLR4 expression, reduced the phosphorylation level of intracellular p38, and decreased the activation of p65 and NF-κB, inhibiting the expression and secretion of the inflammatory cytokines IL-2, IL-6, and TNF-α. Moreover, JH-3 also inhibited the release of cytochrome c and the expression of TNF-αR2, caspase-8, and caspase-9 and reduced the levels of caspase-3, inhibiting *Salmonella*-CVCC541-induced apoptosis of RAW264.7 cells (Figure 8). These findings lay a foundation for the application and development of AMPs and for future studies of the infection-inhibiting activity of JH-3.

## 4. Materials and Methods

### 4.1. Materials

*Salmonella* CVCC541 and RAW264.7 cells were purchased from the China Institute of Veterinary Drug Control (Beijing, China). The peptide JH-3 (purity ≥ 98.0%, RRFKLLSHSLLVTLASHL, 18 amino acid residues, molecular weight 2091.51 kD, aliphatic index 151.67, net charge 12) was synthesized by Shanghai Gil Biochemical Co., Ltd., China and was dissolved in ddH_2_O.

### 4.2. Preparation of RAW264.7 Cells

RAW264.7 cells were cultured in six-well tissue culture plates and incubated until the cell culture formed a single monolayer of cells. Next, the cells were divided into six groups: (1) negative control; (2) cells treated with *Salmonella* CVCC541; (3) cells treated with JH-3 alone; (4) cells treated with *Salmonella* CVCC541 and JH-3 (RAW264.7 cells were incubated with 1 MIC of JH-3 for 1 h prior to the addition of CVCC541 at an MOI of 10); (5) cells treated with LPS and JH-3 (RAW264.7 cells were treated with JH-3 for 1 h, after which LPS was added at a final concentration of 1 μg/mL); and (6) cells treated with LPS at a final concentration of 1 μg/mL. Cells were infected with *Salmonella* CVCC541 that were grown to the logarithmic growth phase (cells were centrifuged at 8000 rpm for 2 min and then were washed twice with 1 × PBS) at a dose of MOI = 10. The cell culture supernatants were collected after 3, 6, 12, and 24 h of infection for ELISA assays of IL-2, IL-6, and TNF-α [35]. In addition, the cells were harvested at 1, 2, 3, 4, 5, and 6 h after infection for qRT-PCR analysis of IL-2, IL-6, and TNF-α [36]. The primer sequences used for qRT-PCR are shown in Table 1.

### 4.3. Evaluation of the Bactericidal Effects of JH-3 in RAW264.7 Cells

RAW264.7 cells (5 × 10^6^) were seeded into 12-well tissue culture plates (NEST, China) and incubated for 12 h at 37 °C with 5% CO_2_. RAW264.7 cells were infected with CVCC541 (MOI = 100) for 2 h. After washing the cells twice, extracellular and surface-bound bacteria were killed by the addition of 50 mg/mL of ciprofloxacin to the culture medium, followed by a 1 h of incubation at 37 °C with 5% CO_2_. After washing the cells twice, the cells were incubated again in Dulbecco’s modification of Eagle’s medium (DMEM) supplemented with JH-3 (1 MIC) or PBS as a control for 1, 2, 3, 4, and 5 h at 37 °C with 5% CO_2_. Finally, the cells were lysed with 100 mL of sterile distilled H_2_O. The cell lysates were subsequently plated on agar and incubated overnight and the colonies were counted [37,38]. 

### 4.4. Western Blot Analysis

RAW264.7 cells were inoculated at a density of 2 × 10^6^ cells per well in six-well tissue culture plates and incubated until the cell culture reached a monolayer. Next, the cells were divided into a negative control group and a group infected with *Salmonella* CVCC541 at an MOI of 10. After infection, RAW264.7 cells were incubated at 37 °C for 0, 1, 2, 3, 4, and 5 h. The total protein was extracted, and the concentration was measured by using a BCA protein assay kit. Next, the total protein was separated by SDS-PAGE and transferred to a polyvinylidene fluoride (PVDF) membrane. The PVDF membrane was blocked with 5% BSA or nonfat milk in PBST and incubated with the primary antibody anti-β-actin (activated caspase-3) at a dilution of 1:1000 in PBST overnight at 4 °C. Subsequently, the membrane was incubated with the secondary antibody at a dilution of 1:5000 in PBST for 1 h at room temperature and then visualized using educational community license (ECL). Each sample was assayed in triplicate. Finally, greyscale analysis was performed to evaluate the protein expression by Image J [39].

### 4.5. Cytotoxicity Detection Assay

Cytotoxicity was measured using an LDH-measuring CytoTox 96 nonradioactive cytotoxicity assay (Roche, batch no. 4744926001). RAW264.7 cells (1 × 10^5^) were inoculated into a 96-well plate and incubated for 12 h and infected with *Salmonella* CVCC541 (MOI = 10 or MOI = 100) or pretreated with JH-3 (1 MIC) for 1 h. LDH detection was performed at 3, 6, 12, and 24 h after infection or with PBS as a negative control. A positive control group was prepared 15 min prior to detection. Total lysis of cells was performed via treatment with 1% Triton X-100 and represented the 100% cytotoxicity positive control. Optical densities at 490 nm were measured using a Power Wave 340 microplate reader (BioTek Instruments Inc., Winooski, VT) and were used to calculate the percentage of cytotoxicity following the manufacturer’s directions. Cytotoxicity was calculated as follows: % cytotoxicity = (cpm experimental − cpm spontaneous)/(cpm maximum − cpm spontaneous) × 100 [12,37].

### 4.6. Apoptosis Assays

RAW264.7 cells were transferred into six-well tissue culture plates at a density of 2 × 10^6^ cells per well and were allowed to grow into a monolayer. After reaching confluence, *Salmonella* CVCC541 in the logarithmic growth phase and JH-3 were added into the cell culture (RAW264.7 cells preparation was the same as Section 2.2). The cells were harvested after 12 h of infection for digestion with 0.25% trypsin. Next, the cells were transferred to 1.5-mL eppendorf tubes (EP) tubes, washed twice with cool PBS, and centrifuged at 800 rpm for 10 min. Subsequently, the cells were mixed with 100 μL of labeling solution (AnnexinV-FITC+PI; the labeled solution was prepared according to the BD manual). Finally, the cells were gently resuspended and incubated at room temperature in the dark for 15–20 min. Each sample was mixed with 400 μL of loading buffer for detection using a flow cytometer [40].

### 4.7. Confocal Laser Scanning Microscopy Assays

For confocal laser scanning microscopy assays, RAW264.7 cells were transferred to cell culture dishes at a density of 2 × 10^5^ cells per well and incubated overnight. Subsequently, the medium was replaced with DMEM without serum and antibody, and the cells were infected with *Salmonella* CVCC541 (MOI = 10). Simultaneously, the JH-3 pretreatment group was prepared (the groups were divided as described in Section 2.2), and the cells were incubated for 2 h. Next, the culture medium was discarded, and the cells were washed three times with 1 × PBS preheated to 37 °C. After removing the PBS, 1 mL of 2.5% glutaraldehyde was added into each well to fix cells overnight at 4 °C. Subsequently, the cell culture dishes were maintained at room temperature for 30 min, after which the fixing solution was removed. The cells were washed three times with 1 mL of PBS (5 min each time) and then were blocked at room temperature for 1 h with 5% nonfat milk in PBS. Subsequently, the cells were incubated with the primary antibody (rabbit anti-caspase-3, -8, -9, TLR4, PI3K, or p-p65) for 1 h. After being washed with 1 × PBS as described above, the cells were incubated with the secondary antibody (fluorescence-488- and 594-labeled goat anti-rabbit IgG) in the dark for 1 h, after which they were washed again as described above and stained with DAPI for 15 min in the dark. After washing, the cells were observed via confocal laser scanning microscopy (Zeiss LSM 780) [41].

### 4.8. Analysis of Cytochrome C Release

Mitochondria were isolated with the Mitochondria Isolation Kit for Mammalian Cells according to the manufacturer’s instructions. Protein concentrations were measured by the bicinchoninic acid (bca) protein assay, and 25 μg of protein was loaded to an sds-page gel and transferred to pvdf, as described above. the primary antibody, mouse monoclonal anti-cytochrome c, was added overnight at 4 °C, and horseradish peroxidase (HRP)-labeled anti-mouse secondary antibody was incubated for 1 h at room temperature. Immunodetection was performed using Amersham^TM^ ECL^TM^ Western blotting detection reagents.

### 4.9. Statistical Analysis

All the tests were performed in triplicate, and significant differences between treatment and control groups were determined by analysis of variance (ANOVA) using SPSS version 18.0 (SPSS, Chicago, IL, USA), with the graphs generated using GraphPad Prism 5.0. “ns” indicates no significant differences *p* > 0.05, “*” indicates a significant difference at *p* < 0.05, “**” indicates a significant difference at *p* < 0.01, and “***” indicates a significant difference at *p* < 0.001 [41].

## 5. Conclusions

In summary, this study demonstrated antimicrobial peptide JH-3 significantly alleviated the damage to macrophages caused by *Salmonella* CVCC541 infection. JH-3 decreased the apoptosis of macrophages and alleviated the cellular inflammatory response. These results suggest that JH-3 may be feasible and promising as a potent antimicrobial agent for the food or animal feed additive industry.

## Figures and Tables

**Table 1:** The primers sequences of IL-2, IL-6, TNF-α, ERK, JNK, p38, Casp-3, Casp-8, Casp-9, and GAPDH for qRT-PCR.

Genes	Sequence	Temperature (°C)
IL-6	F:5’-TGGATGGTCTTGGTCCTTAGCC-3’	58
	R:5’-ACTGATGGTGACAACCACG-3’	
ERK	F:5’-ACCCTGGAAGCCATGAGA-3’	60
	R:5’-TAAGGTCGCAGGTGGTGT-3’	
JNK	F:5’-TATACGCATAAGTACGGCTACA-3’	60
	R:5’-GTCCTGGTGGGAAATGAAC-3’	
p38	F:5’-TCGAGACCGTTTCAGTCCATC-3’	60
	R:5’-GGGTCACCAGGTACACGTCATT-3’	
GAPDH	F:5’-CCTTCCGTGTTCCTACCC-3’	59
	R: 5’-GCCCTCAGATGCCTGCT-3’	
TNF-α	F:5’-GGCAGGTCTACTTTGGAGTCATTGC-3’	58
	R: 5’-ACATTCGAGGCTCCAGTGAATTCGG-3’	
IL-2	F:5’-CTGCAGCGTGTGTTGGATTT-3’	60
	R: 5’-GGCTCATCATCGAATTGGCAC-3’	
Caspase-3	F:5’-AGCTTGGAACGGTACGCTAA-3’	60
	R: 5’-TGCATATGCCCATTTCAGGA-3’	
Caspase-8	F:5’-TGCCTCCTCCTATGTCCTGT-3’	60
	R: 5’-AGAGCTGTAACCTTATCAGAAACA-3’	
Caspase-9	F:5’-CCATGAGAGCTTCGGAGAGAA-3’	58
	R: 5’-ACCTTCCCAGGTTGCCAATG-3’	
TNF-αR1	F: 5′-GCCTCCCGCGATAAAGCCAACC-3′	58
	R: 5′-CTTTGCCCACTTTCACCCACAGG-3′	
TNF-αR2	F: 5′-ACACCCTACAAACCGGAACC-3′	58
	R: 5′-AGCCTTCCTGTCATAGTATTCCT-3′	

## Abbreviations

IL-2	interleukin 2
IL-6	interleukin 6
JH-3	antimicrobial peptide JH-3
MIC	minimum inhibitory concentration
MOI	multiplicity of infection
RAW264.7	macrophage
*Salmonella* CVCC541	*Salmonella enterica* Serovar Typhimurium strain CVCC541
TNF-α	tumor necrosis factor-α
